# A Case of Large Ovarian Tumour in a Young Girl

**Published:** 1892-09

**Authors:** J. Macpherson Lawrie

**Affiliations:** Physician to the Weymouth Sanatorium


### A CASE OF LARGE OVARIAN TUMOUR IN A YOUNG GIRL.

BY

J. MACPHERSON LAWRIE, M.D., C.M. Glasg.,

*Physician to the Weymouth Sanatorium.*

---

Elizabeth C., unmarried, a delicate, fragile girl of 16, was admitted into the Weymouth Sanatorium on the 25th of March, 1892, on account of a gradually increasing enlargement of the abdomen of about two years' duration. Her general health had been fairly good. The menstrual discharge appeared at intervals of about two weeks, and was rather profuse. Her ailment caused her a good deal of discomfort and some pain, and she had become the object of unjust suspicion in her neighbourhood.

On examination, a large, prominent swelling was found, occupying the greater part of the abdominal cavity and extending up to the ensiform cartilage. The circumference of the body at the level of the umbilicus was forty inches. Fluctuation was distinct over a considerable area. The percussion note was dull, except low down in the flanks. On vaginal examination, the fundus uteri, not easily defined, was found pushed over towards the left; and, filling up the right half of the pelvis, an irregular hard swelling could be felt continuous with that in the abdomen.

The operation was performed in the usual way. An incision about three inches long was made in the middle line. Several arteries were caught up and the cyst was exposed without much trouble. Spencer Wells's trocar was introduced at the most prominent part, and a considerable quantity of brown fluid evacuated. The trocar was withdrawn and the aperture in the cyst enlarged. On introducing my hand, the tumour was found to be in great part solid, and it was impossible to accomplish any important reduction in its size. It now became necessary to enlarge the abdominal incision, which was done with scissors in an upward direction, and after a good deal of trouble the tumour was drawn out of the abdomen. On account of its breadth, the pedicle was ligatured in three portions and divided. After cleansing the peritoneal cavity, the edges of the wound were brought together with eleven silk-worm gut sutures, and the operation completed with a dressing of iodoform gauze. The tumour and its contents weighed over twenty-five pounds.

The further progress of the case was satisfactory. The temperature did not rise over 100°, and on the third day was normal. At the end of a week the wound was found healed and the sutures removed, and the patient was quite well in a fortnight.

This case contrasts strongly with that of a patient in the Sanatorium last year, from whom I removed an ovarian tumour, complicated with chronic peritonitis and extensive adhesions.[1] The patient was a married woman, 37 years of age, and the mother of five children. She had noticed for two years a slowly increasing enlargement of the abdomen. An attack of peritonitis supervened and passed away, leaving the general enlargement unaffected. She had two other attacks of peritonitis, in the last of which several quarts of dark-brown fluid were

[1] *Lancet*, 1891, vol. ii., p. 1040.

removed by tapping. About six or seven weeks after this she came under my care, and an ovarian cyst, either unilocular or with one cavity greatly predominating, was diagnosed. At the time of operation, the diseased and thickened peritoneum was recognised. It was found impossible to remove the cyst, owing to strong and numerous adhesions to intestines, bladder, &c. I stitched the edge of the cyst all round to the edge of the abdominal wound, except at one place, through which I passed a glass drainage-tube into the abdominal cavity. Another was passed into the sac. For many days the cavities of the abdomen and sac were washed out with carbolised water. The greater part of the sac sloughed and was removed piecemeal. On the twenty-eighth day there was scarcely any discharge, the wound was almost closed, and the patient was sitting up.

Although the result in both these cases was equally satisfactory, they were associated with very different conditions. In one, there was a history of a rapidly growing tumour, without any adhesions; the operation was completed without a drop of fluid finding its way into the peritoneal cavity and the wound was closed without any provision for drainage. In the other, there was a history of paracentesis, peritonitis, extensive adhesions, and accumulation of fluid in the abdomen. The operation was prolonged, and the outlook seemed hopeless. Every legitimate effort had been made to find a pedicle. The termination of the case affords ground for condemning the imprudence of many operators, who too rashly interfere with adhesions and lose their patients; and it also shows what can be accomplished in even the most unfavourable conditions by thorough drainage.

